# Effects of Annealing Temperature Combinations in InO_x_/AlO_x_ Heterostructure for High-Performance and Stable Solution-Processed Junctionless Transistors

**DOI:** 10.3390/ma18225142

**Published:** 2025-11-12

**Authors:** Jinhong Park, Dohyeon Gil, Se Jin Park, Jae Wook Ahn, Minsu Choi, Philippe Lang, Jaewon Jang, Do-Kyung Kim, Jin-Hyuk Bae

**Affiliations:** 1 School of Electronic and Electrical Engineering, Kyungpook National University, 80 Daehakro, Bukgu, Daegu 41566, Republic of Korea; pjinh953@knu.ac.kr (J.P.);; 2 ITODYS, University of Paris Cité, CNRS UMR 7086, 15 rue Jean-Antoine de Baïf, CEDEX 13, 75205 Paris, France; 3 School of Electronics Engineering, Kyungpook National University, 80 Daehakro, Bukgu, Daegu 41566, Republic of Korea; 4 Department of Advanced AI Engineering, Kangwon National University, Samcheok 25913, Republic of Korea

**Keywords:** metal-oxides, junctionless thin-film transistors, thermal annealing, positive bias instability

## Abstract

Junctionless (JL) thin-film transistors (TFTs) are promising candidates for low-cost, large-area electronic devices, but improvements in mobility and bias stability are still required. In this study, the effects of independent annealing of the indium oxide (InO_x_) channel layer and the aluminum oxide (AlO_x_) capping layer (CL) on the performance and reliability of InO_x_/AlO_x_ heterostructure JL TFTs are examined. Devices were fabricated via solution deposition and photopatterning, and the InO_x_ and AlO_x_ layers were annealed at 250 °C and 400 °C. Increasing the annealing temperature from 250 °C to 400 °C, the InO_x_ layer crystallized and densified. The AlO_x_ layer remained amorphous at both temperatures, but its metal-hydroxyl content decreased with higher annealing. For both layers, JL TFTs annealed at 400 °C exhibited the best electrical performance (threshold voltage = 1.82 ± 0.40 V, subthreshold swing = 0.50 ± 0.07 V dec^−1^, saturation mobility = 1.57 ± 0.37 cm^2^ V^−1^ s^−1^). The threshold voltage shift under positive bias stress was 1.70 V, which demonstrates excellent bias stability. These results show that simultaneous high-temperature annealing of the channel and CL is essential to reduce trap-assisted scattering and stabilize electrostatics in JL TFTs, providing practical process guidelines for bias-stable and high-performance oxide electronics.

## 1. Introduction

Oxide semiconductor (OS)-based thin-film transistors (TFTs) have emerged as key building blocks for next-generation electronics owing to their wide bandgap nature that enables high optical transparency, strong stability under high electric fields, and high breakdown voltage, as well as their inherent advantages of very low leakage current and compatibility with low-temperature (<400 °C) processing [1,2,3,4]. OS technology has successfully replaced hydrogenated amorphous silicon in displays and has become the standard backplane for organic light-emitting diode panels [5]. Beyond displays, OS TFTs show strong potential for back-end-of-line compatible transistor applications in monolithic three-dimensional integration and are increasingly investigated for optoelectronics, sensors, and neuromorphic computing [6,7,8].

OS-based junctionless (JL) TFTs are attracting attention because their architecture simplifies fabrication by eliminating source-drain (S-D) junction formation and reducing the overall process complexity and cost [9,10]. The advantages of JL TFTs become even greater when they are implemented through solution process with solution-compatible, photolithography-free patterning methods. However, conventional OS TFTs are typically fabricated via mature vacuum processes (e.g., sputtering) and conventional photolithography [11,12,13]. These OS TFTs generally exhibit excellent electrical characteristics and reliability. Recent studies have reported field-effect mobility comparable to that of low-temperature polycrystalline silicon TFTs [14,15]. In contrast, OS TFTs fabricated using solution process often exhibit relatively low mobility and reliability [16]. This suggests that while using solution process is attractive to maximize the benefits of JL TFTs, improvements in electrical performance and reliability are still required.

In solution-processed JL TFTs, the channel and conductive S–D regions are defined by the selective formation of an oxide capping layer (CL) that modulates carrier concentration of the channel. Heterostructures such as indium oxide/aluminum oxide (InO_x_/AlO_x_) are promising, as the AlO_x_ CL both passivates the back channel and suppresses excess carriers in the InO_x_ channel [17]. Precise control of carrier concentration in the channel and S–D regions is crucial, since excessive conductivity prevents full depletion under negative gate bias, while overly resistive S–D regions reduce on current and saturation mobility (μ_SAT_) [18]. Therefore, co-optimizing the channel and CL properties (balancing carrier concentration, trap density, and structural quality) is essential. This requires understanding how thermal treatment of semiconductor and CLs affects electron concentration, defect states, and crystallinity, and consequently determines key device parameters such as threshold voltage (V_T_) and subthreshold swing (SS).

In addition, for n-type OS TFT-based display backplanes and integrated driver circuits, robustness under positive bias stress (PBS) is a key performance indicator because the devices are repeatedly subjected to positive gate bias [19]. The threshold voltage shift (ΔV_T_) under bias directly affects pixel current accuracy and lifetime uniformity. Therefore, optimizing the thermal annealing of both the semiconductor layer and the CL in JL heterostructures is crucial for ensuring device reliability [20].

In this study, we systematically examine the effects of annealing combinations of the InO_x_ semiconductor and AlO_x_ CL on the electrical characteristics and reliability of solution-processed InO_x_/AlO_x_ heterojunction JL TFTs. The devices were fabricated using a minimal and efficient process based on solution deposition and photopatterning. The InO_x_ and AlO_x_ layers were each annealed at either 250 °C or 400 °C to generate the target combinations. To clarify how the channel and CL properties affect device behavior, transmission electron microscopy (TEM), X-ray photoelectron spectroscopy (XPS), and X-ray reflectivity (XRR) were employed. The contact and channel resistances were separated using the transmission line method (TLM) to isolate the effect of heat treatment on the resistance components.

The bias stability of the device was then evaluated through PBS test. The results revealed that annealing not only InO_x_ but also AlO_x_ significantly improved μ_SAT_ and reduced SS. Furthermore, simultaneous high-temperature annealing of InO_x_ and AlO_x_ produced the most favorable device characteristics—namely, the highest μ_SAT_, lowest SS, and an appropriate positive V_T_, and the smallest ΔV_T_ under PBS—thereby providing a clear pathway toward high-performance, bias-stable JL TFTs.

## 2. Materials and Methods

### 2.1. Preparation of Oxide Precursor Solutions

InO_x_ and AlO_x_ precursor solutions were prepared for JL TFT fabrication. A 0.1 M InO_x_ solution was synthesized by dissolving indium nitrate hydrate (In(NO_3_)_3_·xH_2_O) in 2-methoxyethanol (2-ME). A 0.3 M AlO_x_ solution was prepared by dissolving aluminum nitrate nonahydrate (Al(NO_3_)_3_·9H_2_O) in 2-ME. All solutions were prepared 24 h before use. The mixtures were stirred at 50 °C for 12 h and then aged under ambient conditions for 12 h to obtain clear, homogeneous solutions.

### 2.2. Fabrication of Devices

Heavily doped p-type silicon wafers with a 100 nm thermally grown SiO_2_ gate dielectric were cleaned by sequential ultrasonication in acetone, isopropyl alcohol, and deionized (DI) water for 10 min each. Ultraviolet (UV)-ozone treatment was then performed to remove organic residues and improve surface hydrophilicity. The InO_x_ solution was spin-coated onto the SiO_2_/p-type Si substrates at 3000 rpm for 20 s. In this configuration, the p-type Si and SiO_2_ serve as the gate and gate insulator, respectively. Patterning of the oxide films was carried out using a water etchant-based photopatterning method previously reported [21]. The substrates were soft-baked at 100 °C for 10 s, followed by UV-ozone treatment through a fine metal mask for 3 min. After exposure, the mask was removed, and the substrates were developed by DI water etching for 1 min. The UV-patterned InO_x_ layers were pre-annealed at 100 °C for 5 min and then post-annealed at either 250 °C or 400 °C for 1 h in ambient air. The AlO_x_ solution was spin-coated onto the InO_x_ layer at 3000 rpm for 20 s. The same photopatterning procedure was used to define the channel regions, with soft-bake and UV exposure times optimized for AlO_x_. Since the concentration and thickness of solution-processed films tend to be proportional, the AlOx layer formed under the same coating and annealing conditions was approximately three times thicker than the InO_x_ layer. The patterned AlO_x_ CLs were pre-annealed at 100 °C for 5 min and then post-annealed at either 250 °C or 400 °C for 1 h in ambient air. Three types of TFTs were fabricated with different heat-treatment combinations. InO_x_ films annealed at 250 °C and 400 °C are denoted as InO-250 and InO-400. Likewise, AlO_x_ films annealed at 250 °C and 400 °C are denoted as AlO-250 and AlO-400. The channel length (L_ch_) and width (W_ch_) of all devices were 100 μm and 500 μm, respectively.

### 2.3. Analysis of Thin Films and Devices

The chemical composition of the InO_x_ and AlO_x_ films was characterized by X-ray photoelectron spectroscopy (XPS, NEXSA, Thermo Fisher Scientific, Waltham, MA, USA) using an Al Kα radiation source (1486.6 eV). For the quantitative analysis of film density, X-ray reflectivity (XRR, ATX-G, Rigaku, Tokyo, Japan) measurements were performed to evaluate the film density of InO_x_ and AlO_x_. For accurate XRR analysis of film density, a film thickness exceeding 5 nm was required. Both 0.3 M indium oxide and 0.3 M aluminum oxide precursor solutions were used to prepare the films. The density values were extracted by fitting the Kiessig fringes in the XRR spectra, providing reliable and quantitative estimation of the mass density based on electron density contrast. The electrical characteristics of the TFTs were measured with a semiconductor parameter analyzer (4200A-SCS, Keithley, Cleveland, OH, USA). All measurements were conducted at room temperature under dark conditions. V_T_ was determined by the constant current method, defined as the gate voltage (V_G_) that induces a drain current (I_D_) = 10 nA × W_ch_ L_ch_^−1^. SS was obtained from the subthreshold region in the current range of I_D_ = 1 to 10 nA, based on the following equation:

(1) $$SS=\frac{{dV}_{G}}{{dlog}_{I_{D}}}$$

The I–V characteristics in the saturation region are described by the following equation [22]:

(2) $$I_{D,sat}=\frac{1}{2}\mu_{SAT}C_{ox}\frac{W}{L}{\left(V_{G}-V_{T}\right)}^{2}$$

C_OX_ denotes gate capacitance per unit area of the insulator layer. μ_SAT_ was extracted at V_G_ = V_T_ + 10 V with the drain voltage (V_D_) = 30 V. The contact resistance (R_C_) and channel sheet resistance (R_SH_) were calculated using the transmission line method (TLM) with the following equation [23]:

(3) $$R_{Total}=\frac{V_{D}}{I_{D}}=\frac{L}{W}R_{SH}+2R_{C}$$

## 3. Results and Discussion

### 3.1. Concept and Operation of JL TFTs

**Figure 1:**
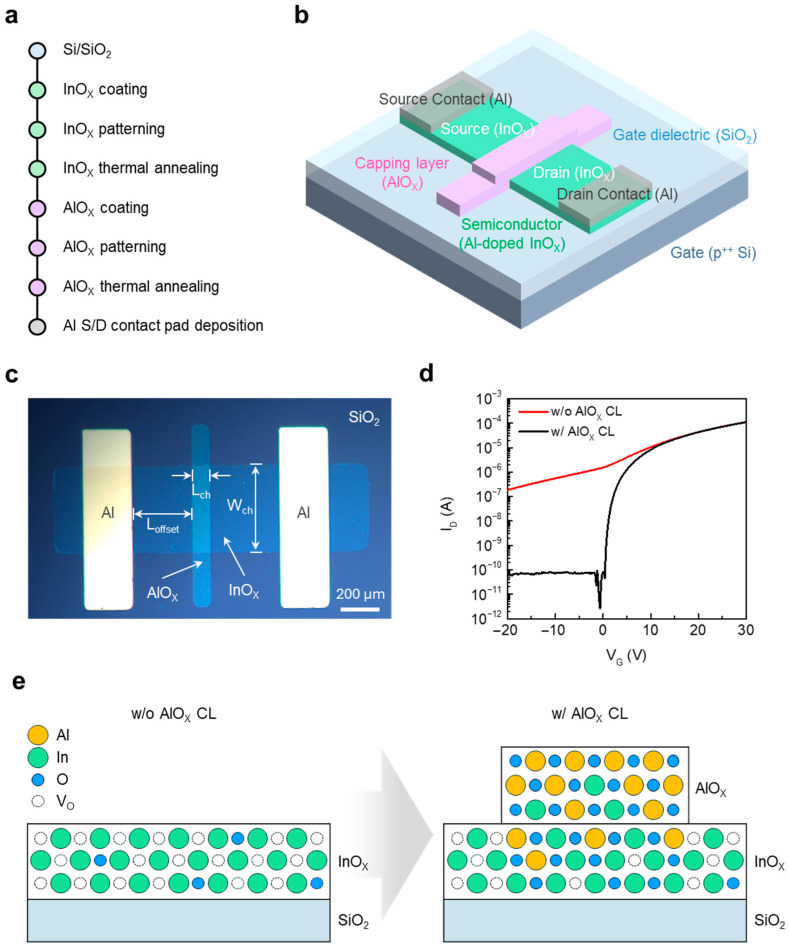
(**a**) Fabrication process flow of solution-processed InO_x_/AlO_x_ JL TFTs. (**b**) Schematic illustration of the fabricated JL TFT. (**c**) Optical microscope image of a representative device, showing the defined L_ch_, W_ch_, and L_offset_. (**d**) Transfer characteristics of JL TFTs with and without the AlO_x_ CL (**e**) Schematic illustration comparing the atomic structure of InO_x_ with and without the AlO_x_ CL.

The device proposed in this study is a JL TFT composed of an InO_x_ semiconductor and an AlO_x_ CL. Figure 1a,b illustrate the fabrication process flow and the device architecture, respectively. Figure 1c presents an optical microscope image of the fabricated JL TFT, where the L_ch_, W_ch_, and offset region (L_offset_) are defined. Similarly to conventional TFT structures, a short L_ch_ is desirable for high on-state current, but short-channel effects must be considered. Furthermore, a short L_offset_ contributes to lowering the contact resistance of the device. In this work, L_ch_, W_ch_, and L_offset_ were fixed at 100, 500, and 400 μm, respectively, for all devices. The transfer characteristics of JL TFTs with and without the AlO_x_ CL are shown in Figure 1d to verify the functional role of the CL. Devices without AlO_x_ exhibited no discernible switching, indicating which confirms that the CL is essential for enabling switching operation. Figure 1e schematically depicts the underlying mechanism. During thermal annealing, Al and O diffuse into the InO_x_ layer, suppressing the electron concentration by reducing the oxygen vacancy (V_O_) density to a level suitable for TFT operation [24]. The observed decrease in electron concentration near the InO_x_/AlO_x_ interface can be attributed to the strong oxygen affinity of Al. During the capping process, partial Al–O interdiffusion occurs, which suppresses oxygen vacancies in the underlying InO_x_ layer. As oxygen vacancies act as electron donors in InO_x_-based oxides, their reduction lowers the free-electron density and shifts the Fermi level toward the mid-gap. Consequently, the conduction-band minimum is raised, resulting in reduced carrier concentration near the interface [25,26,27]. As a result, the InO_x_-only film remains highly conductive and cannot be fully depleted, whereas diffusion from the AlO_x_ CL modulates the conductivity of InO_x_. This CL-driven carrier modulation is the key principle that enables JL TFT operation.

### 3.2. Effects of Annealing Temperature on the Structural and Chemical Characteristics of the InO_x_ Semiconductor and AlO_x_ CL

**Figure 2:**
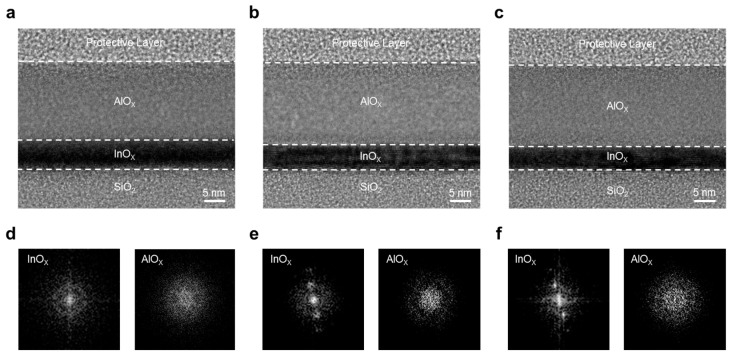
TEM images of the InO_x_/AlO_x_ heterostructure: (**a**) InO-250/AlO-250, (**b**) InO-400/AlO-250, and (**c**) InO-400/AlO-400. Corresponding FFT patterns for (**d**) InO-250/AlO-250, (**e**) InO-400/AlO-250, and (**f**) InO-400/AlO-400.

To investigate the structural effects of annealing temperature on the InO_x_/AlO_x_ stack, cross-sectional TEM analyses were performed. Figure 2a–c show TEM images of InO-250/AlO-250, InO-400/AlO-250, and InO-400/AlO-400 stacked films, respectively. A well-defined bilayer is observed in all cases. The AlO_x_ layer exhibits a relatively uniform, amorphous phase regardless of annealing condition, which is consistent with the known amorphous nature of AlO_x_ even at high annealing temperatures. To assess crystallinity, the corresponding fast Fourier transform (FFT) patterns are shown in Figure 2d–f. In the InO-250/AlO-250 sample, the InO_x_ layer exhibits a diffused halo, which is characteristic of amorphous materials. In contrast, the InO-400/AlO-250 sample exhibits discernible diffraction spots, while the InO-400/AlO-400 sample shows sharper, more distinct spots, indicative of a higher crystalline fraction. These results confirm that InO_x_ undergoes a significant amorphous-to-crystalline transition at 400 °C, forming an ordered matrix that is expected to reduce structural disorder and facilitate charge transport. By comparison, AlO_x_ remains amorphous under all annealing conditions, as corroborated by its FFT halos. The absence of grain boundaries in amorphous AlO_x_ supports its role as a passivation layer that effectively blocks moisture and oxygen from the environment.

**Figure 3:**
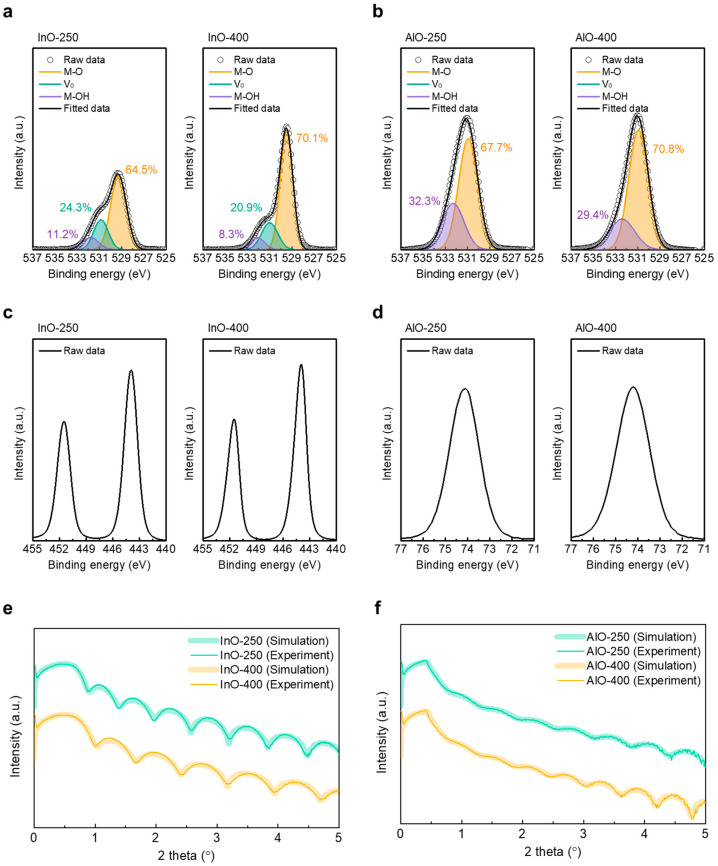
XPS O 1s spectra of (**a**) InO-250 (**left**) and InO-400 (**right**), and (**b**) AlO-250 (**left**) and AlO-400 (**right**). XPS In 3d spectra of (**c**) InO-250 (**left**) and InO-400 (**right**). XPS Al 2p spectra of (**d**) AlO-250 (**left**) and AlO-400 (**right**). XRR spectra of (**e**) InO-250 and InO-400 and (**f**) AlO-250 and AlO-400.

XPS was employed to analyze changes in chemical bonding in InO_x_ and AlO_x_ as a function of annealing temperature. All spectra were calibrated to the C 1s reference at 284.8 eV. The O 1s envelope was deconvolved into three Gaussian components at approximately 530.15, 530.86, and 532.07 eV, corresponding to lattice metal–oxygen bonds (M–O), V_O_, and metal–hydroxide (M–OH), respectively. Figure 3a compares the O 1s spectra of InO-250 (left) and InO-400 (right). The relative fractions of M–O, V_O_, and M–OH were 63.7%, 20.3%, and 15.9% for InO-250 and 70.1%, 20.9%, and 8.3% for InO-400, respectively. These results indicate that high-temperature annealing effectively removes hydroxyl species, thereby strengthening M–O bonding and stabilizing the oxide network. A similar trend was observed in AlO_x_ (Figure 3b), where the M–OH fraction decreased from 32.3% to 29.4% and the M–O fraction increased from 67.7% to 70.8% with higher annealing temperature. Figure 3c,d shows In 3d spectra of InO_x_ and Al 2p spectra of AlO_x_ films, respectively. The In 3d spectrum exhibits two well-defined peaks at approximately 444.5 eV (In 3d_5/2_) and 452.0eV (In 3d_3/2_), corresponding to the spin–orbit doublet characteristic of fully oxidized In^3+^ in InO_x_. No metallic indium component was detected, confirming complete oxidation of the InO_x_ layer. Similarly, the Al 2p spectrum shows a single sharp peak centered at ~74.4 eV, consistent with Al^3+^ in AlO_x_. These results indicate that both InO_x_ and AlO_x_ films maintain stable stoichiometric oxide states without sub-oxide or metallic phases. XRR data are shown in Figure 3e,f for InO_x_ and AlO_x_, respectively. In InO_x_, the density increased from 5.67 g cm^−3^ (InO-250) to 6.19 g cm^−3^ (InO-400), while the thickness decreased from 14.12 nm (InO-250) to 11.43 nm (InO-400) by increasing the annealing temperature. The film thickness, density, and surface roughness of the InO_x_ and AlO_x_ films are summarized in Table 1. These changes are consistent with densification during annealing and align with TEM and FFT evidence of crystallization at 400 °C. By contrast, AlO_x_ exhibited negligible changes in density and thickness between 250 °C and 400 °C, supporting the conclusion that annealing strongly affects InO_x_ crystallinity and densification, while its impact on AlO_x_ is comparatively minor within this temperature range.

### 3.3. Electrical Characteristics of InO_x_/AlO_x_ Heterostructure JL TFTs

**Figure 4:**
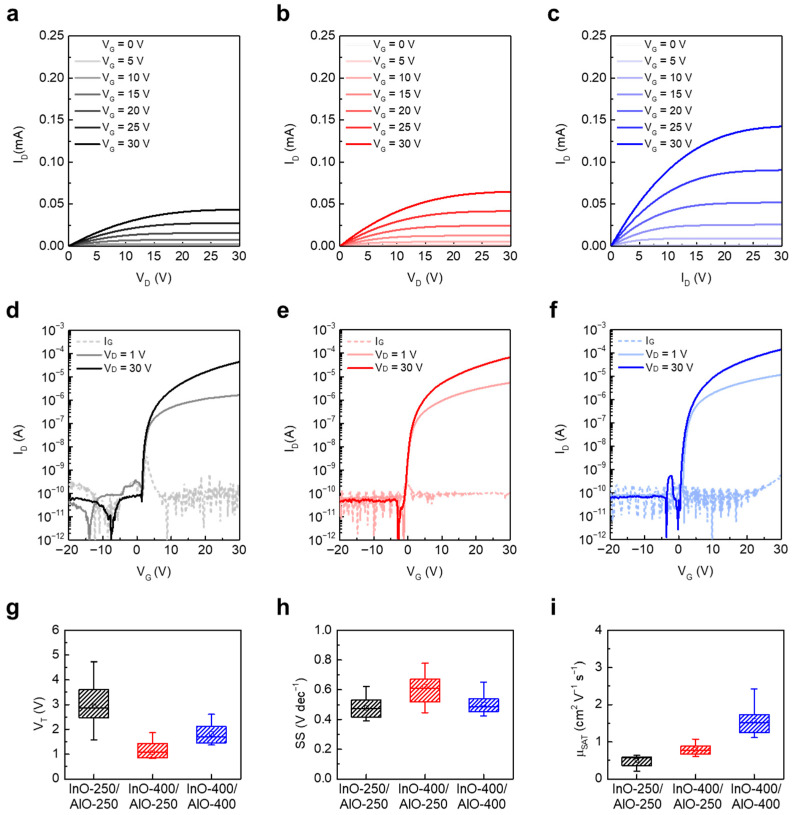
Output characteristics of (**a**) InO-250/AlO-250, (**b**) InO-400/AlO-250, and (**c**) InO-400/AlO-400 JL TFTs. Transfer characteristics of (**d**) InO-250/AlO-250, (**e**) InO-400/AlO-250, and (**f**) InO-400/AlO-400 JL TFTs. Box plots of extracted electrical parameters: (**g**) V_T_, (**h**) SS, and (**i**) μ_SAT_.

To correlate structural and chemical evolution with device operation, the electrical characteristics of the three JL TFT types were evaluated. Figure 4a–c present the output characteristics of InO-250/AlO-250, InO-400/AlO-250, and InO-400/AlO-400 JL TFTs, respectively. For these measurements, V_G_ was varied from 0 to 30 V in 5 V intervals, and V_D_ was swept from 0 to 30 V at a fixed V_G_. All devices exhibited well-defined linear and saturation regions, which confirms proper channel formation. I_D_ increased monotonically with annealing temperature, consistent with enhanced charge transport resulting from denser, crystallized InO_x_ and AlO_x_ films with fewer traps.

Figure 4d–f show the transfer characteristics measured by sweeping V_G_ from −20 to +30 V at fixed V_D_ values of 1 V and 30 V. All devices exhibited low off currents and gate leakage currents on the order of 10^−12^–10^−10^ A, which is attributed to effective patterning and isolation of the active region [28]. Figure 4g–i summarize the extracted parameters. The V_T_ values of InO-250/AlO-250, InO-400/AlO-250, and InO-400/AlO-400 were 2.95 ± 0.89 V, 1.07 ± 0.57 V, and 1.82 ± 0.40 V, respectively (Figure 4g). The largest V_T_ observed in InO-250/AlO-250 is attributed to the nearly amorphous, poorly conductive InO_x_ channels containing deep defects [29]. In contrast, the lowest V_T_ in InO-400/AlO-250 is plausibly linked to the higher electron density of crystallized InO_x_ combined with hydrogen-related states remaining in low-temperature AlO_x_ and insufficient suppression of V_O_ in InO_x_ by AlO_x_ diffusion. Annealing AlO_x_ at 400 °C mitigates –OH–related defects and appropriately reduces electron density, which produces a positive V_T_ shift and a more stable turn-on even with the same InO-400 channel.

The SS values were 0.48 ± 0.07 V dec^−1^ (InO-250/AlO-250), 0.62 ± 0.13 V dec^−1^ (InO-400/AlO-250), and 0.50 ± 0.07 V dec^−1^ (InO-400/AlO-400) (Figure 4h). SS in JL TFTs reflects the combined influence of interface/bulk traps and the depletion capacitance (C_dep_). Although InO-250/AlO-250 exhibits a small SS, thin film analysis does not necessarily indicate a low trap density. Instead, the lower apparent SS likely arises from reduced electron density, which allows for efficient depletion (smaller C_dep_). By contrast, InO-400/AlO-250 shows the largest SS, consistent with the combined effects of higher channel electron density (larger C_dep_) and residual –OH–related traps in AlO-250. The InO-400/AlO-400 device achieves a low SS by combining a crystalline, adequately doped InO_x_ channel with a trap-suppressed AlO_x_ CL. The SS of conventional solution-processed oxide TFTs was at the values of 0.36 to 4.87, and the SS of the junctionless device proposed in this study was at a comparable value [29,30,31].

The μ_SAT_ values were 0.50 ± 0.14, 0.79 ± 0.14, and 1.57 ± 0.37 cm^2^ V^−1^ s^−1^ for InO-250/AlO-250, InO-400/AlO-250, and InO-400/AlO-400 JL TFTs, respectively (Figure 4i). Crystallization and reduced structural disorder from densification of InO_x_ at 400 °C contributed to the improvement in μ_SAT_.

In addition, the suppression of interface scattering and trapping through annealing of AlO_x_ at 400 °C further enhanced μ_SAT_, as reflected in the trends across the three devices. Consequently, the InO-400/AlO-400 device exhibited the highest μ_SAT_. 

### 3.4. Contact and Channel Resistance of InO_x_/AlO_x_ Heterostructure JL TFTs

**Figure 5:**
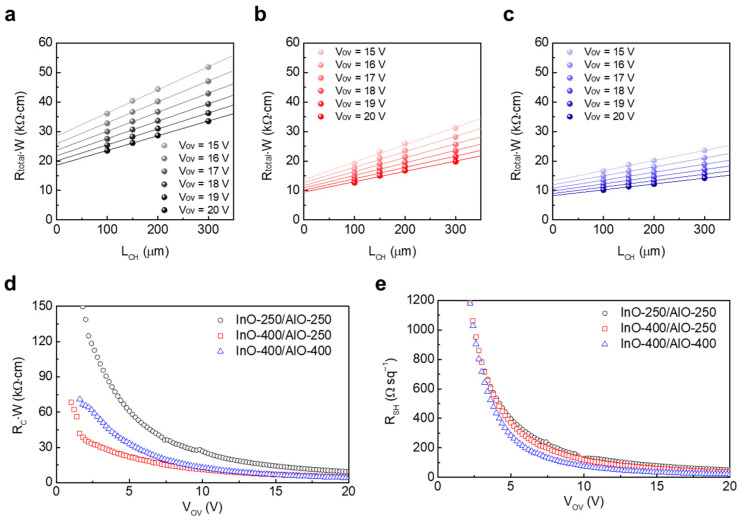
V_OV_-dependent R_Total_·W variations in the (**a**) InO-250/AlO-250, (**b**) InO-400/AlO-250, and (**c**) InO-400/AlO-400 TFTs. Extracted (**d**) R_C_·W and (**e**) R_SH_ of the devices as a function of V_OV_.

The TLM was used to separate the contact and channel contributions. Figure 5a–c show the width-normalized total resistance (R_Total_·W) as a function of L_ch_, for InO-250/AlO-250, InO-400/AlO-250, and InO-400/AlO-400 JL TFTs, respectively. Transfer curves were measured in the linear region (V_D_ = 1 V) for devices with variable channel lengths on the same wafer. The R_Total_·W–L_ch_ plots were highly linear under all conditions. The intercept yields the R_C_·W, and the slope yields R_SH_. As expected, both R_C_·W and R_SH_ decrease with increasing gate overdrive voltage (V_OV_) = V_G_ − V_T_, reflecting the simultaneous mitigation of the metal/semiconductor injection barrier and carrier scattering as the accumulated charge increases.

Among the three types of devices, InO-250/AlO-250 exhibited the largest R_C_·W and R_SH_ at V_OV_ (Figure 5d,e). This is attributed to the amorphous, low-density InO_x_ containing abundant –OH defects, which degrade the channel conduction and simultaneously increase the resistance of L_offset_. These results are consistent with TEM, XPS, and XRR findings. In InO-400/AlO-250, both resistances decreased significantly at 400 °C because crystallization and densification of InO_x_ enhanced injection and bulk transport. However, R_SH_ remained higher than in InO-400/AlO-400, which suggests that residual –OH-related defects in AlO-250 still induced scattering and trapping near the channel. InO-400/AlO-400 devices exhibited the lowest R_SH_ across the entire bias range, and R_C_·W were comparable to (or lower than) those of InO-400/AlO-250, indicating that both channel and contact were optimized simultaneously. These TLM results are consistent with the I-V characteristics in Figure 4. While the high R_C_·W and R_SH_ in InO-250/AlO-250 led to low μ_SAT_, InO-400/AlO-400 achieved the highest μ_SAT_ by reducing both R_C_·W and R_SH_ and suppressing interface traps to maintain a low surface potential.

### 3.5. Positive Bias Stability of InO_x_/AlO_x_ Heterostructure JL TFTs

**Figure 6:**
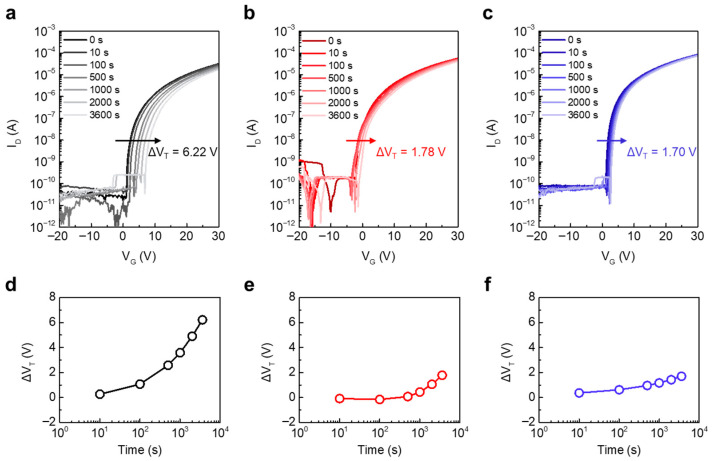
Evolution of the transfer characteristics of (**a**) InO-250/AlO-250, (**b**) InO-400/AlO-250, and (**c**) InO-400/AlO-400 JL TFTs under PBS with V_STR_ of 20 V. Time-dependent ΔV_T_ under PBS of (**d**) InO-250/AlO-250, (**e**) InO-400/AlO-250, and (**f**) InO-400/AlO-400 JL TFTs.

Bias reliability is a critical performance metric for practical applications. We therefore performed PBS tests at a stress overdrive voltage (V_STR_) of 20 V. Figure 6a–c show the transfer curve evolution with stress time for InO-250/AlO-250, InO-400/AlO-250, and InO-400/AlO-400, respectively, and Figure 6d–f summarize the corresponding time dependence of ΔV_T_. The V_T_ of the InO-250/AlO-250 JL TFT gradually shifted in the positive direction, reaching a large ΔV_T_ = 6.22 V. This is attributed to the effective electron trapping caused by the high trap densities of the InO_x_ and AlO_x_ thin films. By contrast, the InO-400/AlO-250 and InO-400/AlO-400 JL TFTs exhibited much smaller final ΔV_T_ values of 1.78 V and 1.70 V, respectively, owing to defect reduction by thermal annealing.

A notable feature in Figure 6e is the non-monotonic ΔV_T_ observed for InO-400/AlO-250. The ΔV_T_ initially shifted negatively (t ≤ 100 s) and then reversed to a positive shift, ultimately converging to 1.78 V. This sign reversal points to the role of low-temperature AlO_x_. Consistent with the XPS results, AlO-250 retained a higher M–OH fraction than AlO-400, which can supply hydrogen-related species. Under positive gate bias, H^+^ can drift toward the interface/channel or be injected into InO_x_, transiently increasing interfacial positive charge or forming shallow hydrogen-related donors. This process produces the initial negative ΔV_T_. As stress continues, electron trapping at deeper states becomes dominant, which drives ΔV_T_ back to positive values. In InO-400/AlO-400 JL TFTs, –OH species were effectively removed, so the H^+^ contribution was minimal. The initial negative component was therefore absent, and the overall ΔV_T_ remained small. Thus, the non-monotonic behavior in Figure 6e serves as a sensitive indicator of hydrogen-related defects in low-temperature AlO_x_ and quantitatively underscores the importance of high-temperature AlO_x_ annealing to ensure PBS stability.

## 4. Conclusions

A systematic study of InO_x_/AlO_x_ JL TFTs fabricated via a solution process was performed to clarify the relationship between processing conditions, film properties, and device performance, which enables device optimization. Independently varying the annealing temperature in the InO_x_/AlO_x_ heterostructure revealed that high-temperature treatment at 400 °C crystallized and densified InO_x_, while suppressing –OH-related interface traps in AlO-400 without altering its amorphous nature. This temperature-dependent understanding of film properties enabled simultaneous optimization of channel transport and contact characteristics. Consequently, InO-400/AlO-400 devices were realized with an optimal configuration. This device demonstrated the following performance parameters: V_T_ = 1.82 ± 0.40 V, SS = 0.50 ± 0.07 V dec^−1^, μ_SAT_ = 1.57 ± 0.37 cm^2^ V^−1^ s^−1^, including the smallest PBS mobility (ΔV_T_ = 1.70 V), and the lowest R_C_·W and R_SH_ under TLM. By contrast, InO-400/AlO-250 exhibited large SS, relatively low μ_SAT_, and non-monotonic ΔV_T_ under PBS due to –OH-related defects in low-temperature AlO_x_, an instability that was resolved by annealing AlO_x_ at 400 °C. These results demonstrate that capping-induced carrier modulation and interface trap control are the key determinants of device performance and stability.

## Figures and Tables

**Table 1:** The film thickness, density, and surface roughness of the InO_x_ and AlO_x_ films.

Samples	Density (g/cm^−3^)	Thickness (nm)	Roughness (nm)
InO-250	5.66693	14.1193	0.7452
InO-400	6.18673	11.4268	0.7080
AlO-250	2.71072	13.3954	0.7961
AlO-400	2.65632	13.9451	0.6466

## Data Availability

The original contributions presented in this study are included in the article. Further inquiries can be directed to the corresponding authors.

## References

[1] Fortunato E., Barquinha P., Martins R. (2012). Oxide Semiconductor Thin-Film Transistors: A review of Recent Advances. Adv. Mater..

[2] Kamiya T., Hosono H. (2010). Material characteristics and applications of transparent amorphous oxide semiconductors. NPG Asia Mater..

[3] Ishibe T., Tomeda A., Komatsubara Y., Kitaura R., Uenuma M., Uraoka Y., Yamashita Y., Nakamura Y. (2021). Carrier and phonon transport control by domain engineering for high-performance transparent thin film thermoelectric generator. Appl. Phys. Lett..

[4] Gong L., Wei M., Yu R., Ohta H., Katayama T. (2022). Significant Suppression of Cracks in Freestanding Perovskite Oxide Flexible Sheets Using a Capping Oxide Layer. ACS Nano.

[5] Park J.S., Maeng W.-J., Kim H.-S., Park J.-S. (2012). Review of recent developments in amorphous oxide semiconductor thin-film transistor devices. Thin Solid Films.

[6] Jang Y., Park J., Kang J., Lee S.-Y. (2022). Amorphous InGaZnO (a-IGZO) Synaptic Transistor for Neuromorphic Computing. ACS Appl. Electron. Mater..

[7] Choi Y., Park W.-Y., Kang M.S., Yi G.-R., Lee J.-Y., Kim Y.-H., Cho J.H. (2015). Monolithic Metal Oxide Transistors. ACS Nano.

[8] Yu X., Marks T.J., Facchetti A. (2016). Metal oxides for optoelectronic applications. Nat. Mater..

[9] Kim J.H., Rim Y.S., Kim H.J. (2014). Homojunction Solution-Processed Meta Oxide Thin-Film Transistors Using Passivation-Induced Channel Definition. ACS Appl. Mater. Interfaces.

[10] Hong S., Na J.W., Lee I.S., Kim H.T., Kang B.H., Chung J., Kim H.J. (2020). Simultaneously Defined Semiconducting Channel Layer Using Electrohydrodynamic Jet Printing of a Passivation layer for Oxide Thin-Film Transistors. ACS Appl. Mater. Interfaces.

[11] Kim H.J., Park K., Kim H.J. (2020). High-performance vacuum-processed metal oxide thin-film transistors: A review of recent developments. J. Soc. Inf. Disp..

[12] Kumomi H., Nomura K., Kamiya T., Hosono H. (2008). Amorphous oxide channel TFTs. Thin Solid Films.

[13] Tückmantel C., Kalita U., Haeger T., Theisen M., Pfeiffer U., Riedl T. (2020). Amorphous indium-gallium-zinc-oxide TFTs patterned by self-aligned photolithography overcoming the GHz threshold. IEEE Electron Device Lett..

[14] Saha J.K., Jang J. (2024). Saturation Mobility of 100 cm^2^ V^–1^ s^–1^ in ZnO Thin-Film Transistors through Quantum Confinement by a Nanoscale In_2_O_3_ Interlayer Using Spray Pyrolysis. ACS Nano.

[15] Wen P., Peng C., Ding X., Chen F.H., Yan G., Xu L., Li J., Li X., Zhang J. (2025). High mobility crystallized stacked-channel thin-film transistors induced by low-temperature thermal annealing. Appl. Phys. Lett..

[16] Ahn B.D., Jeon H.-J., Sheng J., Park J., Park J.-S. (2015). A review on the recent developments of solution processes for oxide thin film transistors. Semicond. Sci. Technol..

[17] Ding Y., Fan C., Fu C., Meng Y., Liu G., Shan F. (2019). High-performance indium oxide thin-film transistors with aluminum oxide passivation. IEEE Electron Device Lett..

[18] Lee K., Kim Y.-H., Yoon S.-M., Kim J., Oh M.S. (2017). Effects of channel thickness on oxide thin film transistor with double-stacked channel layer. J. Korean Phys. Soc..

[19] Conley J.F. (2010). Instabilities in amorphous oxide semiconductor thin-film transistors. IEEE Trans. Device Mater. Reliab..

[20] Zheng S., Wang C., Lv S., Dong L., Li Z., Xin Q., Song A., Zhang J., Li Y. (2025). Enhancement in Performance and Reliability of Fully Transparent a-IGZO Top-Gate Thin-Film Transistors by a Two-Step Annealing Treatment. Nanomaterials.

[21] Kim D.-K., Seo K.-H., Kwon D.-H., Jeon S.-H., Hwang Y.-J., Wang Z., Park J., Lee S.-H., Jang J., Kang I.M. (2022). Viable strategy to minimize trap states of patterned oxide thin films for both exceptional electrical performance and uniformity in sol–gel processed transistors. Chem. Eng. J..

[22] Acharya V., Agarwal K., Mondal S. (2023). Electronic materials for solution-processed TFTs. Mater. Res. Express.

[23] Nguyen C.P.C., Trinh T.T., Raja J., Le A.H.T., Lee Y.-J., Dao V.A., Yi J. (2015). Source/drain metallization effects on the specific contact resistance of indium thin zinc oxide thin film transistors. Mater. Sci. Semicond. Process.

[24] Lee J., Eun J.-S., Na J.-H., Park W., Park J.-H., Feng J., Jang J., Kang I.M., Park J., Zhang X. (2024). Verifying the physical role of upper-active layer on charge transport together with bias stability in bilayer-channel oxide thin-film transistors. Surf. Interfaces.

[25] Kim T.H., Park W., Oh S., Kim S.-Y., Yamada N., Kobayashi H., Jang H.Y., Nam J.H., Habazaki H., Lee B.H. (2021). Unveiling the Role of Al_2_O_3_ Interlayer in Indium-Gallium-Zinc-Oxide Transistors. Phys. Status Solidi A.

[26] Hinuma Y., Gake T., Oba F. (2019). Band alignment at surfaces and heterointerfaces of Al_2_O_3_, Ga_2_O_3_, In_2_O_3_, and related group-III oxide polymorphs: A first-principles study. Phys. Rev. Mater..

[27] Gupta B., Hossain M.A., Sharma A., Zhang D., Tan H.H., Jagadish C., Cathpole K., Hoex B., Karuturi S. (2022). Recent Advances in Materials Design Using Atomic Layer Deposition for Energy Applications. Adv. Funct. Mater..

[28] Yeh C.-C., Zan H.-W., Soppera O. (2018). Solution-Based Micro-and Nanoscale Metal Oxide Structures Formed by Direct Patterning for Electro-Optical Applications. Adv. Mater..

[29] Meng Y., Liu G., Liu A., Song H., Hou Y., Shin B., Shan F. (2015). Low-temperature fabrication of high performance indium oxide thin film transistors. RSC Adv..

[30] Ruzgar S., Caglar Y., Caglar M. (2020). The influence of low indium composition ratio on sol-gel solution-deposited amorphous zinc oxide thin film transistors. J. Mater. Sci.-Mater. Electron..

[31] Hsu C.-C., Chou C.-H., Chen Y.-T., Jhang W.-C. (2019). A Study of Solution-Processed Zinc-Tin-oxide Semiconductors for Thin-Film Transistors. IEEE Trans. Electron Devices.

